# SARS-CoV-2-ORF3a variant Q57H reduces its pro-apoptotic activity in host cells

**DOI:** 10.12688/f1000research.146123.1

**Published:** 2024-04-23

**Authors:** Maria Landherr, Iuliia Polina, Michael W. Cypress, Isabel Chaput, Bridget Nieto, Bong Sook Jhun, Jin O-Uchi

**Affiliations:** 1 Medicine, University of Minnesota Twin Cities, Minneapolis, Minnesota, 55455, USA

**Keywords:** mitochondria, apoptosis, cell death, cell signaling

## Abstract

**Background:**

Mutations in the viral genome of severe acute respiratory syndrome coronavirus 2 (SARS-CoV-2) can enhance its pathogenicity by affecting its transmissibility, disease severity, and overall mortality in human populations. In addition to mutations within the coding region of SARS-CoV-2 structural proteins, there have been reports of mutations in other SARS-CoV-2 proteins that affect virulence, such as open reading frame 3a (ORF3a), which is involved in viral replication. The expression of ORF3a in host cells activates cell death signaling, leading to tissue damage, which affects the severity of COVID-19. The ORF3a-Q57H variant is the most frequent and recurrent variant of ORF3a and is likely associated with increased transmissibility but lower mortality in the 4th epidemic wave of COVID-19 in Hong Kong. Computational structural modeling predicted that the Q57H variant destabilizes the protein structure of ORF3a, which may result in reduced protein expression in human cells. However, it is still unknown how this mutation affects ORF3a protein function and, if so, whether it can change the severity of host cell damage.

**Methods:**

**Plasmids carrying** SARS-CoV-2-ORF3a from Wuhan-Hu-1 strain (i.e., wild-type; WT) and its variant Q57H were transiently transfected into HEK293T cells and used for biochemical and cell biological assays.

**Results:**

SARS-CoV-2-ORF3a-Q57H variant exhibits higher protein expression than WT, but ORF3a-Q57H expression results in less apoptosis in host cells compared to WT via lower activation of the extrinsic apoptotic pathway.

**Conclusion:**

The relatively mild phenotype of the SARS-CoV-2-ORF3a-Q57H variant may result from alterations to ORF3a function by this mutation, rather than its protein expression levels in host cells.

## Introduction

COVID-19 is an infectious disease caused by severe acute respiratory syndrome coronavirus 2 (SARS-CoV-2), which is responsible for the global pandemic that began in 2020. ^1^ SARS-CoV-2 can produce 29 proteins, including 9 accessory proteins encoded by open reading frames. ^2^^,^^3^ These proteins were originally identified as critical factors for viral entry, viral genome production and replication, virion morphogenesis, and viral release from the host cells. ^2^^,^^4^

Mutations in the SARS-CoV-2 genome can alter its pathogenic potential, ultimately affecting the severity and transmissivity of COVID-19 in humans. ^5^ Since 2020, the World Health Organization has been identifying, tracking, characterizing, and labeling some SARS-CoV-2 variants as “variants of interest” and “variants of concern” to prioritize global monitoring and research. ^6^ Thirty-six non-synonymous and 78 synonymous mutations have been reported in open reading frame 3a (ORF3a), which is the largest accessory protein in the SARS-CoV-2 genome. ^7^ The 25563G>T-(Q57H) variant is the most common ORF3a variant (30-40%) reported in COVID-19 patients in the US, and the next most frequent ORF3a variant is 10 times less prevalent than Q57H. ^8^ Q57 is located near the end of the first transmembrane domain of ORF3a, facing the hydrophobic lipid interface, ^9^^,^^10^ which changes the amino acid glutamine (Q), which has a non-charged polar side chain, into the positively charged amino acid histidine (H). ORF3a-Q57H was first identified in Singapore in 2020 and has since been observed in the COVID-19 Beta, epsilon, and Mu variants. ^3^ Q57H was the only mutant consistently reported with a high frequency in the entire period of 2020, whereas the frequency of the other ORF3a mutations fluctuated. ^10^ In the fourth epidemic wave of COVID-19 in Hong Kong, this variant was associated with increased transmission and decreased mortality rates. ^11^ Viral samples isolated from patients during this wave did not exhibit enhanced replication kinetics or cytokine/chemokine induction in the host cells. A recent study using computational modeling ^9^ predicted that the Q57H mutation may decrease protein stability and increase the rigidity of the ORF3a protein compared to the original Wuhan-Hu-1 strain ( *i.e.*, wild-type; WT), which likely affects downstream signaling in host cells. However, it has not been established whether the Q57H mutant affects the function of ORF3, its role in host cell damage during SARS-CoV-2 infection, and ultimately the severity of COVID-19 phenotypes in patients.

Based on the computational prediction of negative folding stability in SARS-CoV-2-Q57H by Wang et al., ^9^ we hypothesized that the SARS-CoV-2-ORF3a-Q57H variant produces less protein expression in host cells than WT-ORF3a, thus exhibiting less oxidative stress and apoptosis, which may contribute to decreased mortality in COVID-19 patients. Here, we report that the SARS-CoV-2-ORF3a-Q57H variant does not exhibit lower protein expression, but rather exhibits a relatively higher expression compared to the WT. Moreover, this variant expression causes less activation of the extrinsic apoptotic pathway in the host cells. Our findings may support the potential molecular linkage between this major mutation and a mild phenotype, but higher transmissibility, in COVID-19 patients.

## Methods

### Plasmids, antibodies, and reagents

The antibodies and plasmids used in the experiments are listed in Tables 1 and 2, respectively. All the cells, chemicals and reagents were purchased from Sigma-Aldrich Corporation (St. Louis, MO, USA) unless otherwise listed in Table 3.

**Table 1.** List of commercial primary antibodies used in this study.

Targeted Protein	Type	Company	Catalog number, RRID	Immunogen
GFP	Mouse monoclonal	Sigma-Aldrich, St. Louis, MO, USA	1181446000, AB_390913	Recombinant *Aequorea victoria* GFP
GFP	Rabbit monoclonal	Cell Signaling Technology, Danvers, MA, USA	2956, AB_1196615	A synthetic peptide corresponding to the amino terminus of GFP
Tubulin	Mouse monoclonal	Sigma-Aldrich	T5168, AB_477579	Sarkosyl-resistant filaments from *S. purpuratus* (sea urchin) sperm axonemes
Mitofusin 2 (Mfn2)	Rabbit polyclonal	Sigma-Aldrich	M6319, AB_477221	Synthetic peptide corresponding to amino acid residues 38-55 of human Mfn2 with C-terminal added cysteine, conjugated to KLH
Cleaved caspase 3 (Asp175)	Rabbit polyclonal	Cell Signaling Technology	9661T, AB_2341188	N-terminal residues adjacent to (Asp175) in human caspase-3
HtrA2/Omi	Rabbit monoclonal	Cell Signaling Technology	9745, AB_11220423	Peptide corresponding to residues surrounding Phe341 of human HtrA2/Omi protein
Optic atrophy-1 (OPA1)	Mouse monoclonal	BD Bioscience, Franklin Lakes, NJ, USA	612607, AB_399889	Amino acids 708-830 from human OPA1
Cyclophilin D (CyD)	Mouse monoclonal	Thermo Fisher Scientific, Waltham, MA, USA	455900, AB_2533820	Recombinant rat Cyclophilin F
Caspase 1 (Cleaved Asp210)	Rabbit polyclonal	Thermo Fisher Scientific	PA599390, AB_2818323	Synthetic peptide corresponding to amino acid residues I280-V299 from human CASP1 (Accession P29466).
LC3 A/B Microtubule-associated protein 1A/1B-light chain 3	Rabbit monoclonal	Cell Signaling Technology	12741, AB_2617131	Peptide corresponding to residues surrounding Leu44 of human LC3B protein (conserved in LC3A)
IL-1β/IL-1F2	Rabbit polyclonal	Novus Biologicals, Centennial, CO, USA	NB600-633, AB_10001060	Recombinant human IL-1β/IL-1F2 produced in *E. coli.*
Glucose-regulated protein with sequence homology to Hsp90 (Grp94)	Rabbit monoclonal	Cell Signaling Technology	20292, AB_2722657	Peptide corresponding to residues surrounding Leu397 of human Grp94
Glucose-regulated protein with sequence homology to Hsp70 (Grp78/Bip)	Rabbit monoclonal	Cell Signaling Technology	3177, AB_2119845	Peptide corresponding to residues surrounding Gly584 of human BiP
C/EBP-homologous protein (CHOP)	Mouse monoclonal	Cell Signaling Technology	2895, AB_2089254	Peptide corresponding to the sequence of human CHOP
NLR family pyrin domain-containing protein 3 (NLRP3)	Rabbit polyclonal	Thermo Fisher Scientific	PA5-79740, AB_2746855	Synthetic peptide corresponding to a sequence at the N-terminus of human CIAS1
Bid	Rabbit polyclonal	Novus Biologicals	NB100-56106SS, AB_2065641	Full-length recombinant mouse Bid protein
Strep tag II	Rabbit polyclonal	GenScript, Piscataway NJ, USA	A00626, AB_915541	Epitope tag peptide NWSHPQFEK conjugated to KLH
Argonaute 2 (Argo2)	Rabbit monoclonal	Cell Signaling Technology	2897S, AB_2096291	Synthetic peptide corresponding to mouse argonaute 2
Cytochrome C	Mouse monoclonal	BD Bioscience	556433, AB_396417	Synthetic peptides of pigeon Cytochrome C

**Table 2.** List of plasmids used in this study.

Inserted gene	Vector Backbone	Source/Provider/RRID (if available)	Company	Notes	Ref.
Mitochondrial matrix-targeted DsRed (mt-RFP)	pDsRed1-N1 (Clontech, Mountain View, CA, USA #6921-1)	Dr. Yisang Yoon			^38^
Empty	pEGFP-C1 (Clonetech)		Clontech		
SARS-CoV-2-ORF3a- P2A-eGFP	pcDNA3.1+P2A-eGFP (GenScript)		GenScript	ORF3a was tagged with GFP by bridging “self-cleaving” small polypeptides (P2A)	
SARS-CoV-2-ORF3a-GFP	pcDNA3.1+C-eGFP (RRID:Addgene_129020)		GenScript		
SARS-CoV-2-ORF3a Q57H-GFP	pcDNA3.1+C-eGFP (RRID:Addgene_129020)		GenScript	This construct was generated by PCR-based site-directed mutagenesis using the SARS-CoV-2-ORF3a-GFP constructs as a template.	
SARS-CoV-2-ORF3a-Q57H-P2A-GFP	pcDNA3.1+P2A-eGFP (GenScript)		GenScript	This construct was generated by PCR-based site-directed mutagenesis using the SARS-CoV-2-ORF3a- P2A-eGFP constructs as a template.	
SARS-CoV-2-orf3a-2xStrep	pLVX-EF1alpha-IRES-Puro (Clontech)	Dr. Nevan Krogan/RRID: Addgene_141383	Addgene, Watertown, MA, USA	Addgene plasmi # 141383,	^39^
Mouse MCU-L-GFP	pEGFP-N1 (Clontech)	Dr. Rosario Rizzuto			^40^
Empty	pLVX-EF1α-IRES-puro		ZAGENO, Cambridge, MA, USA	PVT2308	

**Table 3.** List of Specific cells, chemicals and regents used in this study.

Name of cells, chemical/reagents	Supplier	Catalog number	Notes	Ref.
HEK293T cells	Dr. Keigi Fujiwara, University of Texas, MD Anderson Cancer Center, Houston, TX, USA	N/A	Used in Figure 1.	^13^
H9c2 rat cardiac myoblasts	ATCC, Manassas, VA, USA	CRL-1446	Used in Figures 2- 5.	^12^
Interleukin 1β (IL-1β)/IL-1F2 recombinant protein	R&D Systems, Minneapolis, MN, USA	501-RL	Used in Figure 3A. 100 ng of recombinant IL-1β was used for the positive control for the western blotting.	
Z-LEHD-FMK	ApexBio, Houston, TX,USA	B3233	Used in Figure 5D. Z-LEHD-FMK (PubChem CID: 10032582) was dissolved in DMSO and used for the final concentration of 20 μM.	
Caspase-8 Staining Kit (Red)	Abnova. Taipei City, Taiwan	KA0760	Used in Figure 4A- C. One μL of Red-IETD-FMK (PubChem CID 25108681) was added to 300 μl of cell culture medium and cells were incubated for 30 min at 37°C incubator with 5% CO _2_. The caspase inhibitor Z-VAD-FMK (PubChem SID: 404336810) at 1 μl/ml was added to inhibit caspase activation.	^41^
FuGENE HD	Promega, Madison, WI, USA	E2312	Used in Figures 1- 5. 0.5-3 μg of plasmids and 7 µl of FuGENE HD was added to 100 μL Opti-mem (Thermo Fisher Scientific) at room temperature. The mixture was incubated for 15 min and added to 2 ml cell culture medium.	
Cell lysis buffer	Cell Signaling Technology	9803S	Used in Figures 1- 5. Two hundred μl of 1x Cell lysis buffer were used for each 6-cm dish to harvest protein.	
Fluorescence-conjugated secondary antibodies	LI-COR Biosciences, Lincoln, NE, USA	926-32211 and 926-68020	Used in Figures 1- 5. Secondary antibodies were added in 0.05% PBST (1;20, 000 dilution). The nitrocellulose membrane was incubated with secondary antibody-containing PBST for 1 hr at room temperature.	
NucView® 405 substrates	Biotium, Fremont, CA, USA	10407	Used in Figure 2C and D. PBS containing 2 µM NucView® 405 substrate was treated to the cells at room temperature for 30 min before observation.	^42^
Cell Meter™ Caspase 9 Activity Apoptosis Assay Kit *Red Fluorescence*	AAT Bioquest, Pleasanton CA, USA	22817	Used in Figure 5D. Five μL of 200X Ac-LEHD-ProRed™ stock solution was added to 1 mL of Assay Buffer provided from the manufacturer to make caspase 9 substrate working solution. Cells were incubated with the working solution at room temperature for 1 hr, before observation.	^43^

### Cell culture and transfection

Study protocol was approved by the Institutional Biosafety Committee at University of Minnesota (#2305-41075H). HEK293T and H9c2 cells were maintained in Dulbecco’s modified Eagle’s medium supplemented with 4.5 g/L glucose, 1 mM sodium pyruvate, 1% L-glutamine, 10% fetal bovine serum, 100 U/mL penicillin, and 100 μg/mL streptomycin at 37 °C with 5% CO _2_ in a humidified incubator, transfected with plasmids (0.5-3 μg/3.5-cm dish) using Fugene HD, and used for experiments 48 to 72-hr after transfection. ^12^^,^^13^

### Western blot analysis

Mitochondria-enriched fractions were separated from cytosolic fractions by different centrifugation speeds and dissolved with lysis buffer containing protease inhibitor cocktails and 1 mM phenylmethylsulfonyl fluoride, and subjected to western blotting. ^12^^,^^13^ The immunoreactive bands were visualized, and the whole blotting images for each figure panel ^14^ were obtained using a near-infrared fluorescence imaging system (LI-COR Biotechnology, Lincoln, NE, USA). ^12^^,^^13^

### Live cell imaging

Cells stained with cell-permeable dyes ( Table 3) were observed by an FV3000 confocal microscope (Olympus, Tokyo, Japan) at room temperature. Localization of GFP-tagged proteins was observed in H9c2 cells stably overexpressing mitochondrial matrix-targeted DsRed (mt-RFP) and the colocalization efficiency was estimated using Pearson’s correlation coefficient. ^12^

### Statistics

All data are presented as the mean ± standard error (SEM). Unpaired Student’s t-test and one-way ANOVA followed by Tukey’s post-hoc test were performed for two-group comparisons and multiple comparisons, respectively, with statistical significance defined as a p-value < 0.05.

## Results

**Figure 1.**
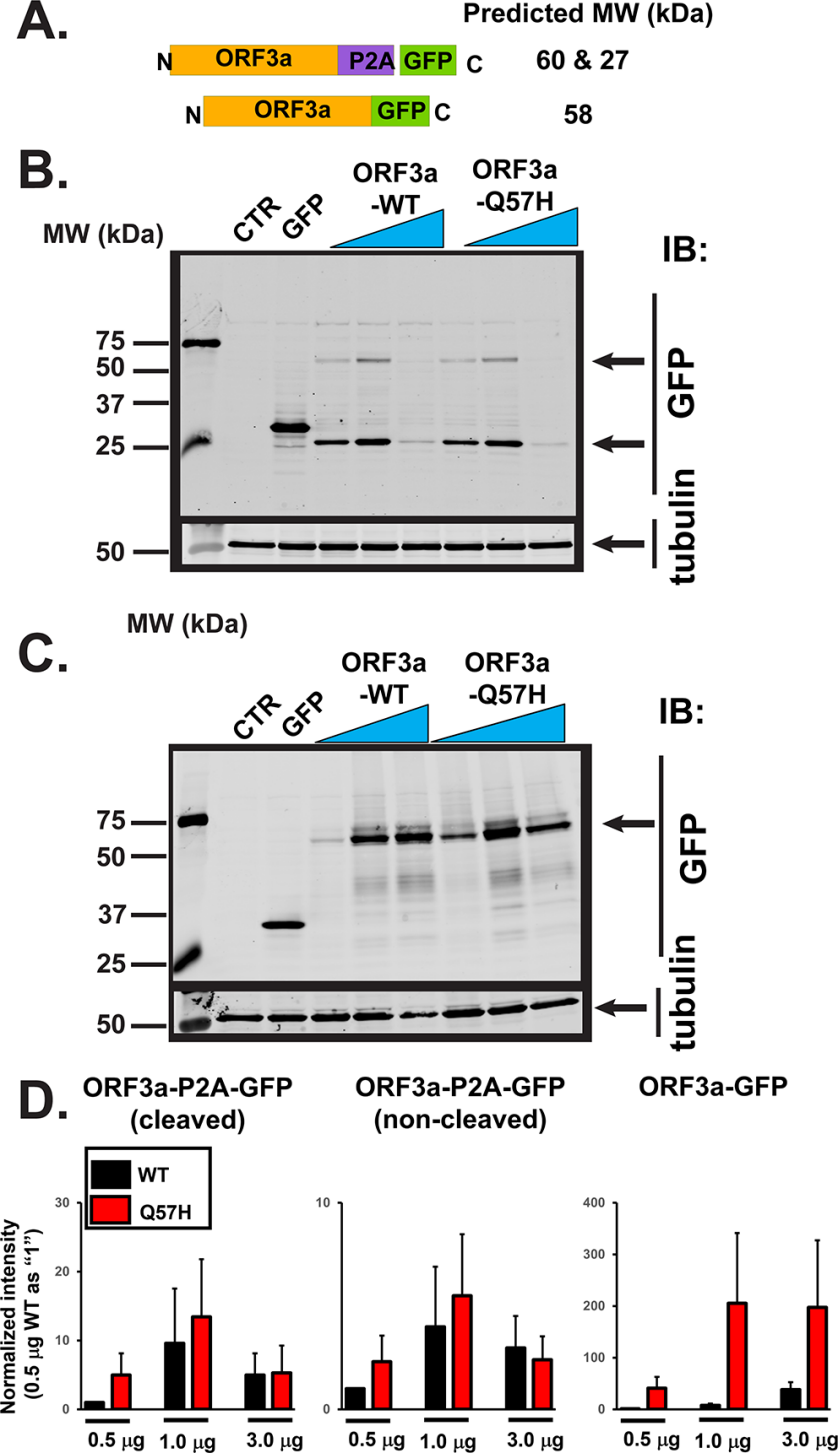
ORF3a-Q57H exhibits higher protein expression compared to WT-ORF3a. A. SARS-CoV-2-ORF3a constructs used in this study. B. Expression of WT- and Q57H mutant ORF3a-P2A-GFP in HEK293T cells. The pEGFP-C1 empty plasmid expressing only EGFP was shown as a control. Each construct was transfected with 0.5, 1.0, or 3.0 μg per 3.5 cm dish. Tubulin was used as a loading control. CTR, cells with no transfection. IB, immunoblotting C. Expression of WT- and Q57H-ORF3a-GFP constructs in HEK293T cells. D. Summary data of B and C (n = 4). In each panel, band intensity was normalized to the value from 0.5 μg of WT- ORF3a construct transfection.

We first tested the effect of Q57H on ORF3a protein expression levels in HEK293T cells by transiently expressing non-tagged and GFP-tagged SARS-CoV-2-ORF3a ( Figure 1A). In the plasmid expressing non-tagged ORF3a, ORF3a is tagged with GFP by bridging “self-cleaving” small polypeptides (P2A), ^15^ allowing for bicistronic expression of non-tagged ORF3a proteins and GFP. Small amounts of the non-cleaved form also existed, but ORF3a protein expression levels were estimated by the amount of cleaved GFP ( Figure 1B). Contrary to computational predictions, ^9^ we did not observe a significant decrease in ORF3a protein expression by Q57H mutations, but rather ORF3a-Q57H exhibited relatively higher protein expression compared to WT-ORF3a ( Figure 1B- D) as assessed by immunostaining with GFP antibody. Thus, differences in protein expression levels between WT and mutant ORF3a are more likely to occur via post-translational processes rather than transcriptional regulation.

**Figure 2.**
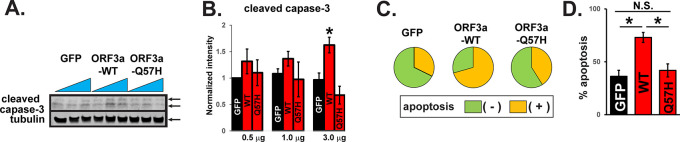
WT-ORF3a but not Q57H activates apoptotic signaling. A. Cleaved caspase-3 in H9c2 cells overexpressing WT and mutant ORF3a. GFP was transfected as a control. Each construct was transfected with 0.5, 1.0, or 3.0 μg per 3.5-cm dish. B. Summary data of A (n= 5). All values were normalized to the value from 0.5 μg of the transfected GFP control. * *p*<0.05. C. Detection of caspase-3 activity in live H9c2 cells stained with Nucview 405 Caspase-3. GFP-positive cells were selected as transfected cells, and nuclear staining-positive cells by fluorogenic DNA dye were counted as apoptotic cells under the confocal microscopy. D. Summary data of C from three independent experiments. * *p*<0.05. N.S., not significant **.**

Using a computational model, Wang et al. predicted that the ORF3a protein structure becomes more rigid and less flexible after the Q57H mutation ^9^ and may result in less activation of downstream signaling that causes host cell damage. We tested cellular damage by WT-ORF3a and Q57H-ORF3a proteins in H9c2 cardiac myoblasts because this cell line is more vulnerable to oxidative, apoptotic, and inflammatory signaling than cancer cell lines, including HEK293T cells. ^12^^,^^16^ We used non-tagged ORF3a constructs ( *i.e.*, ORF3a-P2A-GFP, Figure 1A) for this assay to avoid the potential impact of tag modification on ORF3a protein activity. GFP was used as the control. A recent report has shown that SARS-CoV-2-ORF3a expression can activate apoptotic signaling. ^17^ We found that the expression of WT-ORF3a, but not ORF3a-Q57H, increased caspase-3 activity in H9c2 cells, as assessed by the amount of cleaved caspase-3 ( Figure 2A and B). Caspase-3 activity was also evaluated by live-cell staining with a fluorogenic DNA dye coupled to the caspase-3/7 DEVD recognition sequence (NucView® substrates). GFP itself produced a population of apoptotic cells as reported, ^18^ but WT-ORF3a expression significantly increased the number of apoptotic cells compared to GFP ( Figure 2C and D). The number of apoptotic cells in Q57H cells was similar to that in GFP cells and significantly lower than that in WT-ORF3a cells ( Figure 2C and D).

**Figure 3.**
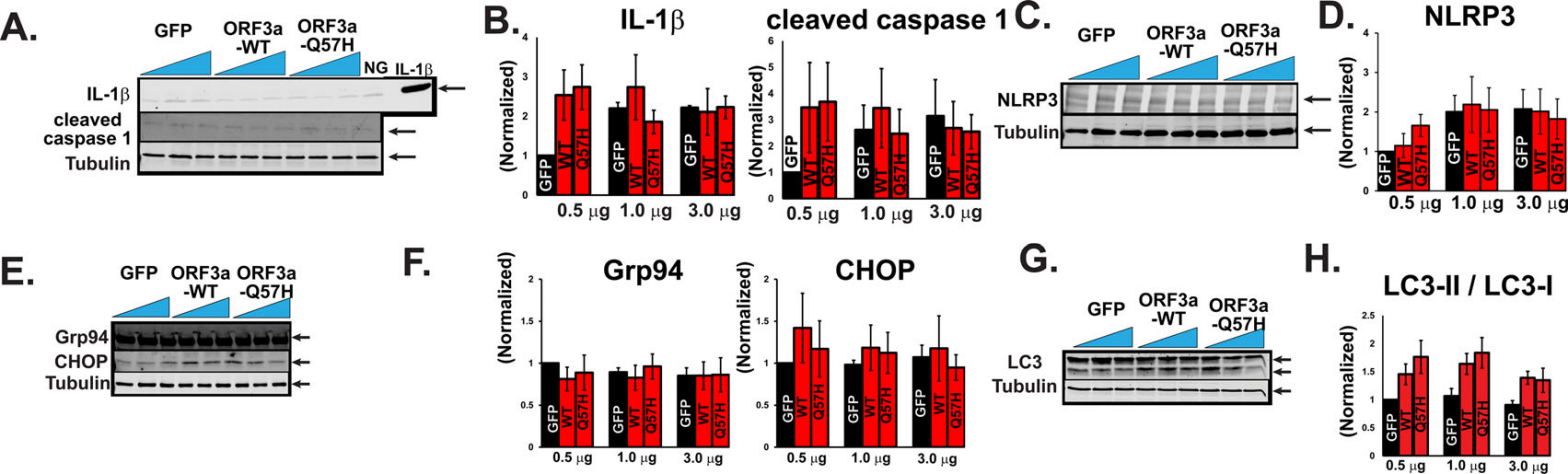
ORF3a-WT and -Q57H do not significantly activate inflammatory, endoplasmic reticulum (ER) stress, and autophagy signaling. A. Assessment of inflammatory activity by cleaved caspase-1 and IL-1β in H9c2 cells expressing WT and mutant ORF3a. GFP was transfected as a control. Cell lysates treated with nigericin (15 μm for 60 min) and a recombinant IL-1β protein were used as positive controls. All values were normalized to the value from 0.5 μg of the transfected GFP control. B. Summary data of A (n=3). C. Detection of the NLRP3 inflammasome in H9c2 cells expressing WT and mutant ORF3a. All values were normalized to the value from the cells transfected with 0.5 μg of GFP. D. Summary data of C (n= 4). E. Assessment of the expression of ER stress markers Grp78, Grp94, and CHOP in H9c2 cells transfected with WT and mutant ORF3a. GFP was transfected as a control. F. Summary data of E (n=4, n =3, n=3, respectively). G. Assessment of autophagic flux by the LC3-II/LC3-I *ratio* in H9c2 cells expressing WT and mutant ORF3a. GFP was transfected as a control. H. Summary data of G (n=5). The ratio of LC3-II (low molecular weight) to LC3-I (high molecular weight) was calculated and normalized to the value from 0.5 ug of the transfected GFP control.

In addition to apoptotic responses, several groups have shown that the expression of SARS-CoV-2-ORF3a constructs with protein tags activate inflammatory signaling, endoplasmic reticulum (ER) stress, and autophagy flux. ^19^^–^^24^ First, non-tagged WT-ORF3a and Q57H did not produce significant inflammatory responses, as assessed by the protein expression levels of IL-1β, NLRP3, and cleaved caspase-1 ( Figure 3A- D). The expression of ER stress markers, including glucose-regulated protein 94 (Grp94), glucose-regulated protein 78 (Bip/Grp78), and C/EBP-homologous protein (CHOP), ^25^ did not change after the expression of either WT-ORF3a or -Q57H in our system ( Figure 3E and F). Finally, both WT-ORF3a and its mutant Q57H showed a similar tendency of increased microtubule-associated protein light chain 3 (LC3)-II/LC3-I ratio, a standard marker indicating the induction of autophagy, but these changes were not significant compared to control cells transfected with GFP ( Figure 3G and H). In summary, SARS-CoV-2-ORF3a expression induces apoptotic signaling activation rather than modulating inflammation, ER stress, and autophagic signaling cascades. Importantly, Q57H expression was less involved in apoptotic signaling activation than that of WT-ORF3a.

**Figure 4.**
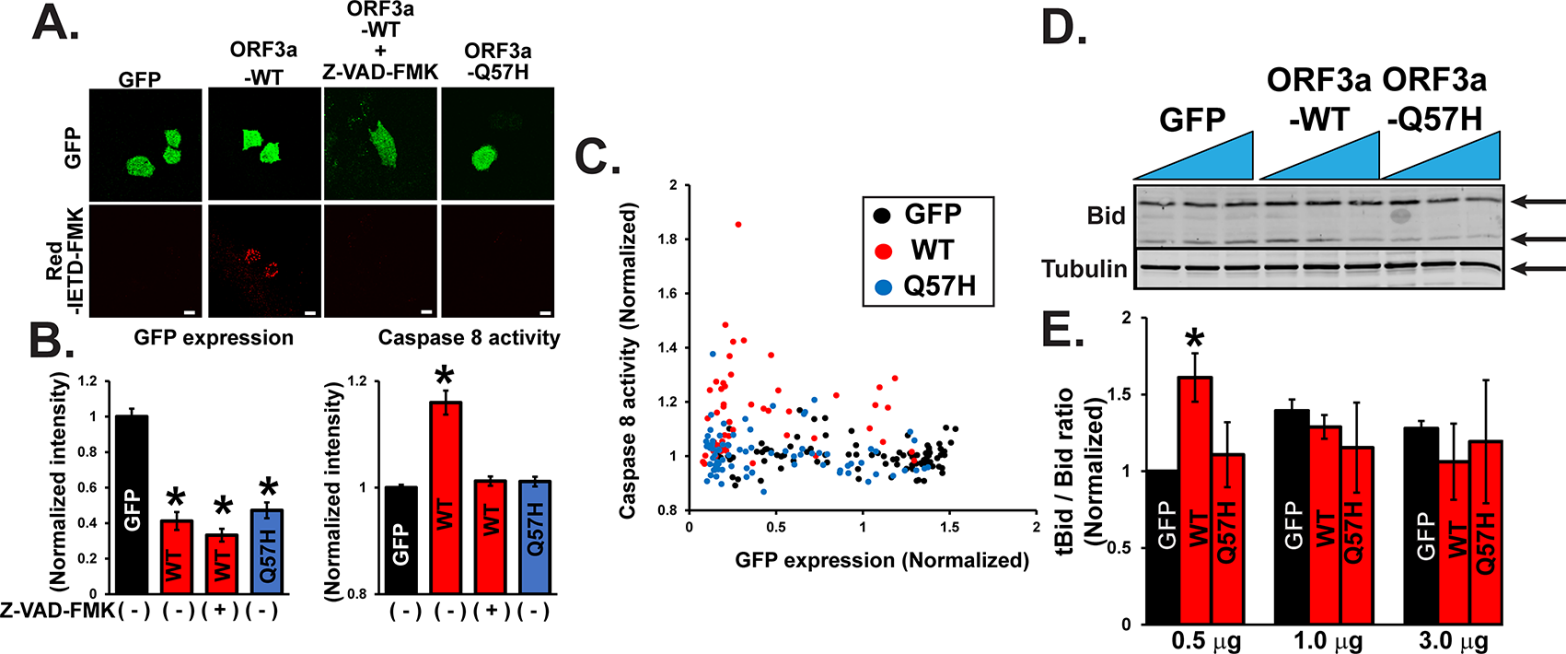
ORF3a-Q57H exhibits less activation of extrinsic apoptotic signaling compared to WT. A. Representative confocal images of H9c2 cells transfected with the indicated plasmids and stained with a cell-permeable marker dye for caspase-8 activation, Red-IETD-FMK. Red-IETD-FMK was detected under confocal microscopy with excitation and emission wavelengths of 488 and 570 nm, respectively. ORF3a-WT overexpressed cells pretreated with Z-VAD-FMK for 1 hr were used as a negative control. Scale Bars = 20 μm B. Summary data of A. * *p*<0.05, compared to GFP-transfected cells. Each fluorescence value was normalized to the average fluorescence calculated from GFP-transfected cells. C. Scatter plots of GFP and Red-IETD-FMK measured from individual cells. D. Representative immunoblot of tBid/Bid in H9c2 cells expressing WT and mutant ORF3a. GFP was transfected as a control. E. Summary data of D. * *p*<0.05, compared to 0.5 ug of the transfected GFP control. * *p*<0.05.

A recent report showed that SARS-CoV-2-ORF3a-ORF3a could activate both the intrinsic and extrinsic pathways of apoptosis. ^17^ Therefore, we examined the activities of signaling molecules from both apoptotic pathways after WT-ORF3a or ORF3a-Q57H expression. The extrinsic pathway caspase-8 was significantly activated by WT-ORF3a expression compared to that in control cells, as assessed by a cell-permeable caspase-8 activity marker, Red-IETD-FMK. However, preincubation with a general caspase inhibitor, Z-VAD-FMK, abolished this change ( Figure 4A and B). Q57H expression did not show significant caspase-8 activation ( Figure 4A and B), and this difference between ORF3a-WT and -Q57H was unlikely based on the expression levels of the constructs, as confirmed by the expression levels of bicistronically expressed GFP ( Figure 4B and C). Caspase-8 is activated by an extrinsic pathway (e.g., cell-surface death receptors) and is known to propagate the apoptotic signal either by directly cleaving and activating downstream caspases (e.g., caspase-3) or by cleaving Bid. ^26^ Cells expressing WT-ORF3a (but only in the lower transfection conditions) showed a significant increase in the truncated Bid (tBid)/Bid ratio, but not by Q57H ( Figure 4D and E). These results suggest that the Q57H variant exhibits less activation of the extrinsic apoptotic pathway compared to the WT.

**Figure 5.**
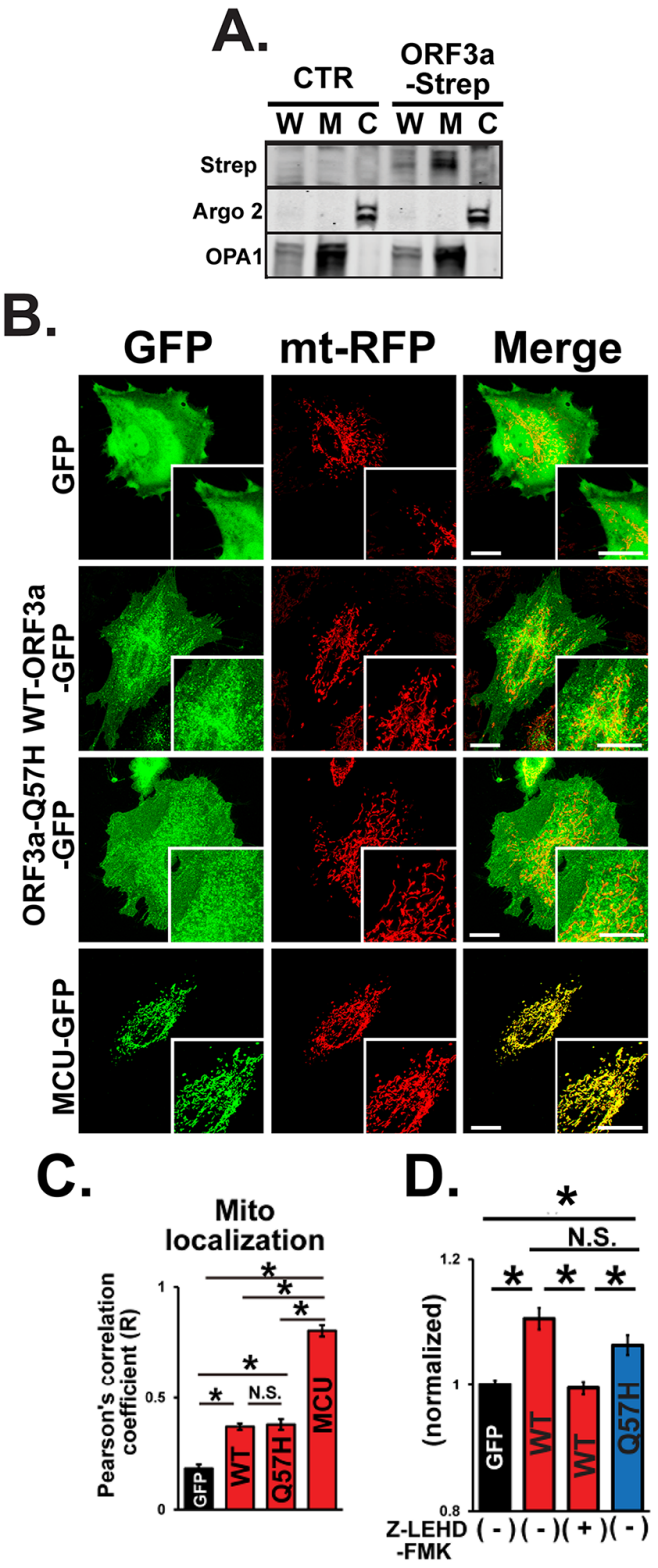
Both WT-ORF3a and ORF3a-Q57H variant activate intrinsic apoptotic signaling. A. Expression of WT-ORF3a-Strep in fractionated proteins from H9c2 cells. Cells transfected with pLVX-EF1α-IRES-puro were used as a control (CTR). Argonaute 2 (Argo2) and optic atrophy-1 (OPA1) were used as markers for the cytosolic fraction (C) and mitochondrial fraction (M), respectively. Whole cell lysates (W) were shown for comparison. B. Representative confocal images of the subcellular localization of GFP, WT-ORF3a-GFP, ORF3a-Q57H-GFP, mitochondrial Ca ^2+^ uniporter (MCU)-GFP (as a positive control) in live H9c2 cells stably expressing mt-RFP. Scale bars = 20 μm. C. Summary data of the mitochondrial localization of GFP constructs estimated by Pearson’s correlation values between the GFP and mt-RFP signals. *p<0.05. N.S., not significant. Cells transfected with a mitochondrial protein MCU-GFP were used as a positive control. D. Assessment of caspase-9 activity in live H9c2 cells transfected with indicated plasmids stained with a cell-permeable caspase-9-specific fluorogenic substrate, Ac-LEHD-ProRed. ORF3a-WT overexpressed cells pretreated for 1 hr with a caspase 9-specific inhibitor, Z-LEHD-FMK, were used as a negative control. ProRed cleaved from Ac-LEHD-ProRed was detected using confocal microscopy with excitation and emission wavelengths of 540 and 620 nm, respectively. The ProRed fluorescence value was normalized to the average fluorescence calculated from GFP-transfected cells.

Next, we investigated the effect of WT-ORF3a and ORF3a-Q57H on the intrinsic apoptotic pathway. Since the SARS-CoV-2-ORF3a protein is predicted to possess three transmembrane domains similar to SARS-CoV-1-ORF3a, and its subcellular localization is likely distributed to several membrane structures, ^27^ we next tested whether ORF3a can be expressed in the mitochondria. Indeed, ORF3a protein was found in the mitochondria-enriched fraction compared to that in the cytosolic fraction ( Figure 5A). Both WT-ORF3a and Q57H-ORF3a were partially localized in the mitochondrial area labeled by mt-RFP, and their subcellular distribution patterns were not significantly different, as assessed by the values of Pearson’s correlation coefficient ( Figure 5B and C). We also found that the Q57H variant was capable of activating caspase-9, and assessed caspase-9 activity, an initiator of intrinsic apoptosis, whose level was comparable to that in WT assessed by Ac-LEHD-ProRed staining ( Figure 5D). In summary, these results suggest that the different caspase-3 activation levels in WT-ORF3a and Q57H-ORF3a are mainly due to their different effects on the extrinsic apoptotic pathway.

## Discussion

**Figure 6.**
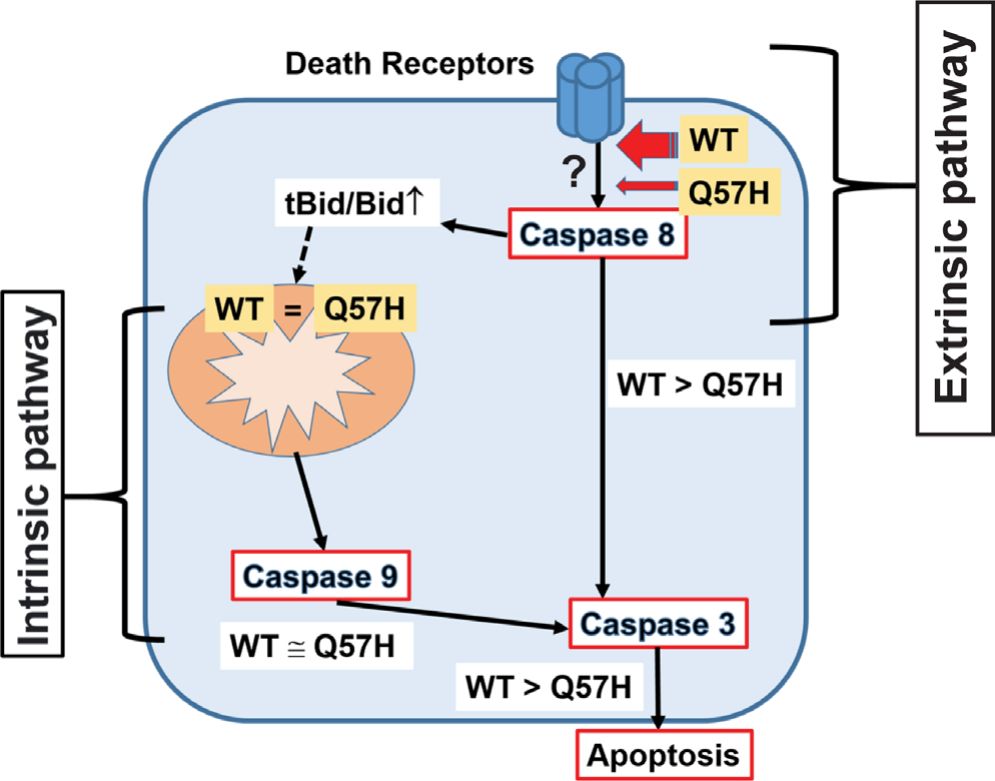
Current Model: SARS-CoV-2-ORF3a-Q57H causes less apoptosis via less activation of the extrinsic apoptotic pathway. SARS-CoV-2-ORF3a induces apoptosis in host cells via activating both intrinsic and extrinsic pathways. ORF3a-Q57H shows less apoptotic activity compared to WT via less activation of the extrinsic apoptotic pathway.

Although the protein sequences of ORF3a from SARS-CoV-1 and CoV-2 have only moderate homology (72%), ^3^ the expression of both proteins in mammalian cells promotes apoptosis. ^17^ Our results showed that Q57H, the most frequent and recurrent variant of SARS-CoV-2-ORF3a, exhibits higher protein expression compared to SARS-CoV-2-ORF3a-WT ( Figure 1) but induces less apoptosis in host cells due to a lack of extrinsic apoptotic pathway activation ( Figures 2- 4, and 6). This property may provide advantages for the SARS-CoV-2-Q57H infection to be relatively mild, thus allowing the virus to have higher transmissivity, as was the case in the fourth epidemic wave of COVID-19 in Hong Kong. ^11^

Although the SARS-CoV-2-ORF3a protein can induce apoptotic signaling activation similar to SARS-CoV-1-ORF3a, ^28^ Ren et al. recently reported that SARS-CoV-2-ORF3a has a relatively weaker effect on activating apoptotic signaling than SARS-CoV-1. ^17^ Moreover, they showed that plasma membrane localization of ORF3a is required for activating apoptotic signaling in SARS-CoV-2. Ren et al. suggested that 1) SARS-CoV-2-ORF3a mainly activates the extrinsic apoptotic pathway, and 2) the intrinsic pathway is secondarily activated downstream of the extrinsic apoptotic pathway. Importantly, the location of the Q57H mutation in the ORF3a structure is far from the key motifs for plasma membrane sorting (i.e., cysteine-rich motif C130/133 and/or tyrosine-based sorting motif Y160 ^17^). This indicates that the mutation does not likely interfere with the plasma membrane sorting of the RFF3a protein, although neither SARS-CoV-2-ORF3a-WT nor -Q57H showed specific plasma membrane expression localized in multiple cellular compartments, including the cytosol and mitochondria ( Figure 5B).

Another key finding was that SARS-CoV-2-ORF3a-WT was able to activate the extrinsic apoptotic pathway in the absence of death receptor ligands ( Figures 4 and 6). This observation ^17^ suggests that it is likely that ORF3a at the plasma membrane is capable of 1) transactivating death receptors (DRs) by direct or indirect interactions with DRs at the plasma membrane, 2) causing conformational changes in the death-inducing signaling complex (DISC) (i.e., association of the receptor-bound Fas-associated cytoplasmic death domain [FADD] and caspase-8), ^29^ and/or 3) inhibiting the activity of cellular FADD-like IL-1β-converting enzyme-inhibitory proteins such as c-FLIP. ^29^ Because Q57H is located near the end of the first transmembrane domain of ORF3a, which is close to the cytoplasmic face, ^9^^,^^10^ the Q57H mutation may alter the interaction between ORF3a, DRs, and/or DISC within or beneath the plasma membrane. Further studies are required to identify the detailed molecular mechanisms by which ORF3a activates DRs and/or DISC, and whether the Q57H mutation alters this mechanism.

Our data also showed that WT-ORF3a and ORF3a-Q57H both activated intrinsic apoptotic signaling at similar levels, even though Q57H exhibited less activation of extrinsic apoptotic signaling compared to WT ( Figures 4- 6). This result indicates that SARS-CoV-2-ORF3a can initiate intrinsic apoptotic signaling independent of extrinsic apoptotic signaling ( Figure 6). In both SARS-CoV-1 and -CoV-2, ORF3a has three predicted transmembrane domains ^3^^,^^10^ and has been localized in several cellular membrane structures/organelles in host cells, including the plasma membrane, endoplasmic reticulum, Golgi, and lysosomes. ^30^^–^^34^ Our protein fractionation and imaging data showed that ORF3a was also localized in the mitochondria ( Figure 5A- C), where it likely increased mitochondrial membrane permeability and promoted the release of apoptotic proteins. SARS-CoV-1-ORF3a can form K ^+^-permeable viroporins ^28^^,^^34^ that are required to induce ORF3a-mediated cell apoptosis. ^28^ Although still controversial, ^33^ SARS-CoV-2-ORF3a might also form K ^+^-permeable channels at the inner mitochondrial membrane (IMM), which can depolarize the mitochondrial membrane potential similar to the opening of endogenous K ^+^ channels expressed at the IMM, such as the mitochondrial BK _Ca_ channel. ^35^ If ORF3a is expressed in the outer mitochondrial membrane (OMM), it is possible that ORF3a may interact with structural proteins that regulate OMM permeability.

Lastly, we tested whether SARS-CoV-2-ORF3a can modulate autophagy flux, ER stress, and inflammatory signaling in addition to apoptosis, but these signaling pathways were not significantly activated in our system ( Figure 3). The different results may be partly due to the use of different cell types, which may provide different ORF3a expression levels and/or sensitivity to the stress-signaling pathway. In addition, the majority of published data ^19^^–^^24^^,^^36^ were generated from ORF3a constructs with various protein tags, which may alter ORF3a protein function because it is a relatively small protein (~30 kDa). Nevertheless, our results clearly showed a major difference in the activation of apoptotic signaling between ORF3a-WT and Q57H, especially in the extrinsic signaling pathway.

In summary, despite its relatively higher protein expression compared to WT, SARS-CoV-2-ORF3a-Q57H variant expression causes less apoptosis in mammalian cells because of lower activation of the extrinsic apoptotic pathway. As our experiments were performed only in cultured cell lines transfected with a part of SARS-CoV-2 (i.e., ORF3a), we still need to consider that our findings cannot be directly applicable to the *in vivo* situation with SARS-CoV-2 infection. Animal models using SARS-CoV-2 are indispensable for exploring the detailed role of the ORF3a signaling pathway *in vivo.* Despite these limitations, our results suggest that the relatively mild phenotype of the Q57H variant observed in 4th epidemic wave of COVID-19 in Hong Kong and several COVID-19 variants (i.e., Beta, Epsilon, and Mu) may result from weaker pro-apoptotic signaling. Assessing the cellular effects of ORF3a mutations will improve our understanding of the pathophysiology of COVID-19 and inform the design of new therapeutic strategies to prevent and treat COVID-19 and its long-term symptoms. ^37^

## Data Availability

Figshare: Supplementary materials for manuscript “SARS-CoV-2-ORF3a variant Q57H reduces its pro-apoptotic activity in host cells”. https://doi.org/10.6084/m9.figshare.24803106.v1. ^14^ This project contains the following underlying data: Original Western blotting images Data are available under the terms of the Creative Commons Zero “No rights reserved” data waiver (CC0 1.0 Public domain dedication).
