# Supplementary material for: SARS-CoV-2-ORF3a variant Q57H reduces its pro-apoptotic activity in host cells
Source: F1000Res. 2025 Nov 22;13:331. Originally published 2024 Apr 23. [Version 2] doi: 10.12688/f1000research.146123.2 (PMC12754350; doi:10.12688/f1000research.146123.2)
Supplement: Peer review history

# Reviewer report: Reviewer response for version 2

**DOI:** 10.5256/f1000research.190658.r435123

**Published:** 2025-12-30

**Authors:** Wei Liu

**Affiliations:** 1 Henan Agricultural University, Zhengzhou, China

I have carefully examined the revised manuscript and the authors' response to my earlier review comments.

   The authors have provided comprehensive and satisfactory responses to all the questions and concerns I raised previously. Corresponding revisions have been appropriately implemented throughout the manuscript, effectively addressing the issues identified in the initial review.

   Upon a detailed re-evaluation of the revised version, I have not found any new scientific flaws, logical inconsistencies, or technical inadequacies.

   I therefore recommend the manuscript for publication without further revisions.

Is the work clearly and accurately presented and does it cite the current literature?

Yes

If applicable, is the statistical analysis and its interpretation appropriate?

Yes

Are all the source data underlying the results available to ensure full reproducibility?

Partly

Is the study design appropriate and is the work technically sound?

Yes

Are the conclusions drawn adequately supported by the results?

Partly

Are sufficient details of methods and analysis provided to allow replication by others?

Yes

Reviewer Expertise:

Virus infection and immunity; Interaction between viruses and host cells

I confirm that I have read this submission and believe that I have an appropriate level of expertise to confirm that it is of an acceptable scientific standard.

# Reviewer report: Reviewer response for version 2

**DOI:** 10.5256/f1000research.190658.r435124

**Published:** 2025-11-25

**Authors:** Julian Leibowitz

**Affiliations:** 1 Microbial Pathogenesis and Immunology, Texas A&M School of Medicine, Bryan, Texas, USA

This revised manuscript is significantly improved from the prior submission and is now approved.  I have no further comments.

Is the work clearly and accurately presented and does it cite the current literature?

Yes

If applicable, is the statistical analysis and its interpretation appropriate?

Yes

Are all the source data underlying the results available to ensure full reproducibility?

Yes

Is the study design appropriate and is the work technically sound?

Partly

Are the conclusions drawn adequately supported by the results?

Partly

Are sufficient details of methods and analysis provided to allow replication by others?

Yes

Reviewer Expertise:

Replication and pathogenesis of coronaviruses

I confirm that I have read this submission and believe that I have an appropriate level of expertise to confirm that it is of an acceptable scientific standard.

# Reviewer report: Reviewer response for version 1

**DOI:** 10.5256/f1000research.160166.r290352

**Published:** 2024-06-27

**Authors:** Wei Liu

**Affiliations:** 1 Henan Agricultural University, Zhengzhou, China

In their manuscript, Landherr et al. showed that SARS-CoV-2-ORF3a variant Q57H reduces its pro-apoptotic activity in host cells. As a consequence, SARS-CoV-2-ORF3a-Q57H variant does not exhibit lower protein expression, but rather exhibits a relatively higher expression compared to the WT. Finally, the authors demonstrated that SARS-CoV-2-ORF3a-Q57H variant expression causes less activation of the extrinsic apoptotic pathway in the host cells. This study adds may support the potential molecular linkage between this major mutation and a mild phenotype. Despite the interesting phenotypic observations, several major issues must be addressed before further consideration. 

 1) The writing lacks clarity and logical coherence, and these issues must be thoroughly resolved to enhance the manuscript's overall quality and scientific rigor. For instance, the sentence “we hypothesized that the SARS-CoV-2-ORF3a-Q57H variant produces less protein expression in host cells than” in the final part of the Introduction, is more appropriate to move this hypothetical sentence in the Discussion.

 2)- In Figure 1B, there are two GFP indicator arrow? In addition, no trend of increasing concentration was observed. The WB-blot need to be repeated.

 3)- In Figure 1D, the band intensity should be was normalized to the value from Tubulin. 

 4)- In Figure 2A, the molecular weight of the caspase-3 should be displayed.

 5)- In Figure 3, the molecular weight of the proteins should be displayed. Figure 3A, the “cleaved” IL-1β should be shown in the panel. Figure 3G, although statistical analysis shows no significant difference in LC3-II to LC3-I, the he expression of LC3-II suggests that there is the activation in t ORF3a-WT and -Q57H. Maybe more representative WB-blot need to be displayed.

 6)- In Figure 4, the tBid or Bid need to be indicated clearly in panel.

 7)- “caspase-8” “Caspase 8” are inconsistent in manuscript.

Is the work clearly and accurately presented and does it cite the current literature?

Yes

If applicable, is the statistical analysis and its interpretation appropriate?

Yes

Are all the source data underlying the results available to ensure full reproducibility?

Partly

Is the study design appropriate and is the work technically sound?

Yes

Are the conclusions drawn adequately supported by the results?

Partly

Are sufficient details of methods and analysis provided to allow replication by others?

Yes

Reviewer Expertise:

Virus infection and immunity; Interaction between viruses and host cells

I confirm that I have read this submission and believe that I have an appropriate level of expertise to confirm that it is of an acceptable scientific standard, however I have significant reservations, as outlined above.

# Response

**Published:** 2025-10-31

**Authors:** Jin O-Uchi

**Affiliations:** Molecular Pharmacology and Phy, University of South Florida Department of Molecular Pharmacology and Physiology, Tampa, Florida, USA

We appreciate the constructive comments from the reviewers and editor. We have addressed all of them in our revisions.

**1) The writing lacks clarity and logical coherence, and these issues must be thoroughly resolved to enhance the manuscript's overall quality and scientific rigor. For instance, the sentence “we hypothesized that the SARS-CoV-2-ORF3a-Q57H variant produces less protein expression in host cells than” in the final part of the Introduction, is more appropriate to move this hypothetical sentence in the Discussion.**

**A: **Following the reviewer’s suggestion, we moved this sentence to the revised discussion section.

**2) In Figure 1B, there are two GFP indicator arrow? In addition, no trend of increasing concentration was observed. The WB-blot need to be repeated.**

**A: **The two arrows indicate the cleaved form (corresponding to the size of GFP) and non-cleaved (the size of ORF3a + P2A + GFP) form of ORF3a-P2A-GFP. The reviewer is correct that we could not observe a concentration-dependent increase in GFP expression from the transfection of either ORF3a-P2A-GFP or ORF3a-Q57H-P2A-GFP. Following another reviewer’s suggestion, we changed our assessment method to use an anti-ORF3a antibody. We have now observed a concentration-dependent increase in ORF3a protein levels. We included ths new data set as a revised Figure 1. The original Figure 1 using anti-GFP antibody has been moved to the online Supplementary Materials as a Supplementary Figure (see https://doi.org/10.6084/m9.figshare.30473513.v1).

**3) In Figure 1D, the band intensity should be normalized to the value from Tubulin**. 

**A:** The band intensity was normalized to the value from tubulin The 0.5 µg GFP/tubulin was set as 1 and showed as relative values in each panel (See online Supplementary Figure 1D). We also clarified all the normalization procedures in each panel in the main figure set

**4) In Figure 2A, the molecular weight of the caspase-3 should be displayed**.

**A:** Added. See also the supplementary materials  https://doi.org/10.6084/m9.figshare.30473513.v1

**5-1) In Figure 3, the molecular weight of the proteins should be displayed.**

**A:** Added. See also the supplementary materials  https://doi.org/10.6084/m9.figshare.30473513.v1

**5-2) Figure 3A, the “cleaved” IL-1β should be shown in the panel.**

**A:** Added “cleaved”.

**5-3) Figure 3G, although statistical analysis shows no significant difference in LC3-II to LC3-I, the expression of LC3-II suggests that there is the activation in the ORF3a-WT and -Q57H. Maybe more representative WB-blot need to be displayed.**

**A:** We performed three additional independent experiments and analyzed a total of 8 experiments. We confirmed that there is still no significant difference. New representative images were shown. See also the supplementary materials  https://doi.org/10.6084/m9.figshare.30473513.v1

**6) In Figure 4, the tBid or Bid need to be indicated clearly in panel.**

**A:** Added the label for tBid to Figure 4 C.

**7) “caspase-8” “Caspase 8” are inconsistent in manuscript.**

**A:** Revised and made consistent with caspase-8 in the revised manuscript.

# Reviewer report: Reviewer response for version 1

**DOI:** 10.5256/f1000research.160166.r290354

**Published:** 2024-06-24

**Authors:** Julian Leibowitz, Sarisa Na Pombejra

**Affiliations:** 1 Microbial Pathogenesis and Immunology, Texas A&M School of Medicine, Bryan, Texas, USA

In this manuscript Landherr et al compare the effect of the common SARS-CoV-2 ORF3a Q57H mutation to the parental sequence on host cells using ectopic over expression strategies.  The manuscript presents the interesting finding that over expression of the ORF3a Q57H protein decreases apoptosis relative to that observed by the parental protein, and that this difference is due to greater activation of the extrinsic apoptosis pathway by the parental protein.  These data are the strength of the manuscript.  However, there are several critical issues not adequately explained or addressed.  Below are specific comments and suggestions to improve the manuscript.

 Specific Comments

 1. Differences in ORF3a Protein Expression:

 -The expression strategy employed is somewhat unusual, in creating an ORF3a-p2A-GFP fusion construct in which the primary translation product undergoes efficient self-cleavage mediated by its picornavirus p2A protease domain, liberating GFP and ORF3a-p2A polypeptides.  This allows tracking transfected cells by GFP fluorescence and the comparison of ORF3a-p2A expression between variant and parental proteins indirectly by performing immunoblots for GFP and quantitating the amount of the fusion proteins and liberated GFP.  They report that, contrary to a computational prediction by Wang et al that the Q57H variant will be less stable than the parental protein, that it is somewhat more stable.  A weakness of this approach is it ignores the possibility that the p2A-GFP fusion to ORF3a has a stabilizing effect on the fusion protein.  Furthermore, by utilizing immunoblots to GFP it makes the assumption that liberated ORF3a-p2A peptides from parental and Q57H variants degrade at equivalent rates and that GFP immunoblots reflect the amount of ORF3a-p2A protein.  It would have strengthened the paper to have directly measured the relative amounts of ORF3a-p2A protein with an anti-ORF3a antibody.

 -The statement "Differences in protein expression levels between WT and mutant ORF3a are more likely to occur via post translational processes rather than transcriptional regulation" (Results, Paragraph 1).   It is not clear what the relevance of transcriptional regulation in transfected cells is when expression of the ORF3a mRNA is regulated quite differently in the context of SARS-CoV-2 infected cells than in transfected cells. Even in the context of transfected cells this claim would need experimental evidence, such as qRT-PCR for transcriptional analysis, post-translational modification studies, or protein folding studies.

 2. Differing Results From Previous Studies:

 -The results negative related to ORF3a effects on inflammasome activation, ER stress, and autophagy in this paper differ from other reported studies.  The authors should discuss potential reasons for these discrepancies.  One obvious difference regarding inflammasome activation is the use of H9c2 cells, a cardiomyoblast cell line, in the current work.  It is likely that these cells are not competent for inflammasome activation since Figure 3A shows that pharmacologic activation of inflammasomes by Nigericin fails to activate inflammasomes (IL-beta or caspase-1 cleavage) in these cells.

 3. Mechanistic Studies on Q57H Mutation:   

 -The manuscript lacks detailed mechanistic studies on how the Q57H mutation could affect extrinsic apoptosis pathway activation.  For example, the authors should have examined the effect of individual death receptor knockdown on ORF3a induction of the extrinsic pathway for apoptosis to both identify the receptor activated and confirm their results.  Additionally, the authors should investigate and discuss the potential alterations in ORF3a interaction with death receptors, and any differences in downstream signaling activation between WT and Q57H. Comparative studies between WT and Q57H regarding their interactions and signaling pathways would significantly enhance the manuscript's contribution to the field.

 Conclusion:

 The manuscript presents interesting findings but requires substantial improvements in experimental support and detailed mechanistic studies. Addressing the points mentioned above will strengthen the manuscript and provide a more comprehensive understanding of the ORF3a protein's role and its mutations.

Is the work clearly and accurately presented and does it cite the current literature?

Yes

If applicable, is the statistical analysis and its interpretation appropriate?

Yes

Are all the source data underlying the results available to ensure full reproducibility?

Yes

Is the study design appropriate and is the work technically sound?

Partly

Are the conclusions drawn adequately supported by the results?

Partly

Are sufficient details of methods and analysis provided to allow replication by others?

Yes

Reviewer Expertise:

Replication and pathogenesis of coronaviruses

We confirm that we have read this submission and believe that we have an appropriate level of expertise to state that we do not consider it to be of an acceptable scientific standard, for reasons outlined above.

# Response

**Published:** 2025-10-31

**Authors:** Jin O-Uchi

**Affiliations:** Molecular Pharmacology and Phy, University of South Florida Department of Molecular Pharmacology and Physiology, Tampa, Florida, USA

We appreciate the constructive comments from the reviewers and editor. We have addressed all of them in our revisions.

**(It would have strengthened the paper to have directly measured the relative amounts of ORF3a-p2A protein with an anti-ORF3a antibody.)**

**A:** Following the reviewer’s suggestion, we used an anti-ORF3a antibody instead of an anti-GFP antibody to re-evaluate the expression levels of WT-ORF3a and ORF3a-Q57H mutant, and replaced the previous data obtained using the GFP antibody with this new data (see Revised Figure 1A-C). When the high amounts of plasmid (≥1.0 µg/well *) *were transfected, ORF3a-WT and -Q57H constructs produced similar levels of ORF3a protein, as assessed with ORF3a-specific antibody (see revised Figure 1B and C). However, under lower amounts of plasmid transfection (0.5 µg/well), we found a significantly higher protein expression of Q57H compared to WT-ORF3a (see revised Figure 1B and C). Based on this new data, we have revised the abstract, result section, and discussion section.

**(It is not clear what the relevance of transcriptional regulation in transfected cells is when expression of the ORF3a mRNA is regulated quite differently in the context of SARS-CoV-2 infected cells than in transfected cells. Even in the context of transfected cells this claim would need experimental evidence, such as qRT-PCR for transcriptional analysis, post-translational modification studies, or protein folding studies.)**

**A:** The qRT-PCR experiment was conducted, and the results were shown in the revised Figure 1D. We found no significant differences in mRNA levels between ORF3a-WT and -Q57H in all transfection conditions. In response to the reviewer’s suggestion, we removed the statement "Differences in protein expression levels between WT and mutant ORF3a are more likely to occur via post-translational processes rather than transcriptional regulation” from the result section.

**(2. The results negative related to ORF3a effects on inflammasome activation, ER stress, and autophagy in this paper differ from other reported studies.  The authors should discuss potential reasons for these discrepancies.  One obvious difference regarding inflammasome activation is the use of H9c2 cells, a cardiomyoblast cell line, in the current work.  It is likely that these cells are not competent for inflammasome activation since Figure 3A shows that pharmacologic activation of inflammasomes by Nigericin fails to activate inflammasomes (IL-beta or caspase-1 cleavage) in these cells.)**

**A:** We used the established protocol (Using the higher concentration of nigericin (NG)  with longer stimulation, see PMID: 34432650) and confirmed that NG can indeed activate inflammatory signaling in H9c2 cells as reported previously (see revised Figure 3A). Nevertheless, following the reviewer’s comment, we added the following sentence to the discussion section: “H9c2 cells may be less competent for inflammasome activation”.

**(-The manuscript lacks detailed mechanistic studies on how the Q57H mutation could affect extrinsic apoptosis pathway activation.  For example, the authors should have examined the effect of individual death receptor knockdown on ORF3a induction of the extrinsic pathway for apoptosis to both identify the receptor activated and confirm their results.  Additionally, the authors should investigate and discuss the potential alterations in ORF3a interaction with death receptors, and any differences in downstream signaling activation between WT and Q57H. Comparative studies between WT and Q57H regarding their interactions and signaling pathways would significantly enhance the manuscript's contribution to the field.)**

**A:** Following the reviewer’s suggestions, we further investigated the potential molecular mechanism underlying the ORF3a-mediated apoptosis and  its alteration by the Q57H mutation. Although we did not screen the ORF3a interaction with all eight types of human death receptors due to the lack of reliable antibodies for co-immunoprecipitations, we found that ORF3a (but not Q57H) increases the protein expression of FADD, a crucial adaptor protein that acts as a bridge between death receptors and caspases to trigger extrinsic apoptotic pathways. The previous report showed that ORF3a expression at the plasma membrane (PM) is required for activating the apoptotic signaling (PMID: 32555321), ORF3a expression at the PM may enhance the stability of FADD protein within the death-inducing signaling complex (DISC) located just beneath the PM. Moreover, we found that the protein expression level of Q57H at the PM is lower thsan that of WT, which may contribute to the reduced activation of the extrinsic apoptotic pathways. We have included this new data in Figure 4D and E and revised the result section, figure legends, and discussion section accordingly.
